# Environmental chemicals in dog testes reflect their geographical source and may be associated with altered pathology

**DOI:** 10.1038/s41598-021-86805-y

**Published:** 2021-04-01

**Authors:** Rebecca N. Sumner, Andrew Byers, Zulin Zhang, Jorgen S. Agerholm, Lena Lindh, Gary C. W. England, Richard G. Lea

**Affiliations:** 1 School of Veterinary Medicine and Science, The University of Nottingham, Nottingham, UK; 2 Environmental and Biochemical Sciences, The James Hutton Institute, Aberdeen, UK; 3 Department of Clinical Veterinary Sciences, University of Copenhagen, Copenhagen, Denmark; 4 Department of Production Animal Medicine, University of Helsinki, Helsinki, Finland

**Keywords:** Developmental biology, Environmental sciences, Pathogenesis

## Abstract

In humans and dogs, a temporal decline in semen quality and increased incidence of testicular cancer is hypothesised to be associated with exposure to anthropogenic chemicals, particularly during fetal development. Human studies suggest that differential exposures to environmental chemicals may be associated with geographical differences in male reproductive health. Here we investigate testicular chemical profiles and pathologies in dogs residing in the UK [West Midlands (WM), East Midlands (EM), South East (SE)], Denmark (Copenhagen) and Finland (Vantaa). Testes, surplus from routine castrations, contained region specific differences in relative concentrations of diethylhexyl phthalate (DEHP), polybrominated diphenyl ethers (PBDE) and polychlorinated biphenyls (PCB). Relative to UK regions, testes from dogs living in Finland and Denmark had higher concentrations of PBDE and lower concentrations of DEHP and PCBs. Regional differences in the UK in PCB concentrations were also observed. Dog testes from Finland had fewer pathologies, reduced testicular area stained for Sertoli and germ cells and evidence of reduced cellular proliferation. Since the geographical differences in testis pathologies in dogs parallel reports of regional differences in human testicular cancer, we postulate that this may reflect chemical effects within the testis and that this may be related to environmental influences on male reproductive function.

## Introduction

For more than seven decades, the presence of anthropogenic chemicals in the environment and their potential effects on human male fertility has been of significant concern. A series of widely cited meta-analyses have reported a temporal decline in human male semen quality and, over the same period, both increased incidences of testicular germ cell cancer in younger men and malformations of babies at birth (cryptorchidism, hypospadias) have been reported^1–3^. This has led to the hypothesis that these abnormalities are linked to exposure to chemical pollutants and that these developmental and functional abnormalities have the same environmental origin^4^. Despite the many studies suggesting that temporal changes in male reproductive function reflect a deleterious effect of environment, true cause and effect remains to be demonstrated. Nevertheless, one may predict that if temporal changes in environmental factors are responsible for the time-based changes in male reproductive function, it is likely that geographical differences in contaminating chemicals may similarly be linked to regional differences in fertility and reproductive function.

In support of this contention, temporal declines in semen quality appear specific to industrialised parts of the world such as North America, Europe and Asia^3^. This is exemplified by regional differences in sperm quality across China, and by reports of younger men in Denmark having poorer sperm quality and a higher incidence of testicular cancer than similar populations in Finland^5,6^. Although attributed to environmental contaminants, few studies have investigated geographical influences on pollutants, particularly those in biological tissues and fluids. Chemical contaminants have however been detected in urine, serum and, to a lesser extent, in seminal plasma^7^. With respect to the latter, some studies report that seminal chemical concentrations negatively correlate with semen quality whereas others indicate that due to the low concentrations present, establishing such a relationship is difficult^8^.

The linkage of post-natal reproductive problems with earlier developmental events, some of which occur during fetal development, suggests that identified testicular pathologies may be a presage of later functional perturbations. For example, male fetuses from pregnant ewes exposed to mixtures of chemicals in a commonly used fertiliser (biosolids), exhibit reduced numbers of Sertoli and Leydig cells^9^. In addition, a subset of male offspring exposed to such fertilizers pre and post-natally exhibit pathologies in their testes such as Sertoli cell-only tubules^10^. Although the weight of evidence suggests that such early pathologies are linked to perturbed reproductive function in the adult, further work is required to consolidate this hypothesis.

In the human, testicular germ cell tumours have been linked to perturbed gonadal development in fetal life and the increased incidence of type 2 testicular germ cell tumours in younger men is reported to show variation with ethnic and geographical origin^4,11,12^. Both environmental and genetic factors have been implicated and some epidemiological studies indicate a link between specific chemical compound types and testicular cancer, e.g., dichlorodiphenyltrichloroethane (DDT) and polychlorinated biphenyls (PCBs)^12,13^. Testicular germ cell tumours are thought to be derived from germ cell neoplasia in situ cells (GCNIS) that reside in the gonad prior to birth^14^. However, the mechanisms underlying the linkage between chemical exposures and testicular cancer remain uncertain^15^. Notably, despite the epidemiological studies described above, no studies have looked at chemical content of testicular tissue as an index of environmental exposure and at indices of histological and/or pathological abnormalities in these same samples.

We have previously shown that a population of stud dogs, in an assistance dog breeding programme, exhibit similar temporal trends in declining semen quality and increasing incidences of cryptorchidism in the offspring, as reported in the human^16^. Since these dogs live in family homes and are therefore exposed to the same household contaminants as their owners, we have previously hypothesised that temporal changes in dog and human reproductive health reflects a common environmental exposure^17^. Indeed, by virtue of sharing our environment for many years, the dog may be a sentinel species for human exposures to environmental household contaminants. In support of this contention, our previous studies have shown that chemicals are present in dog testicular tissue and seminal plasma at concentrations able to inhibit sperm motility in vitro^16,18^. Given the availability of testes from dogs undergoing routine castration for veterinary purposes, the present study was designed to use the dog as an index (sentinel) species of human exposure and to determine if dog testes from different geographical locations exhibit differences in chemical profiles and pathology.

## Results

### Relative quantification of tissue pathologies

**Figure 1:**
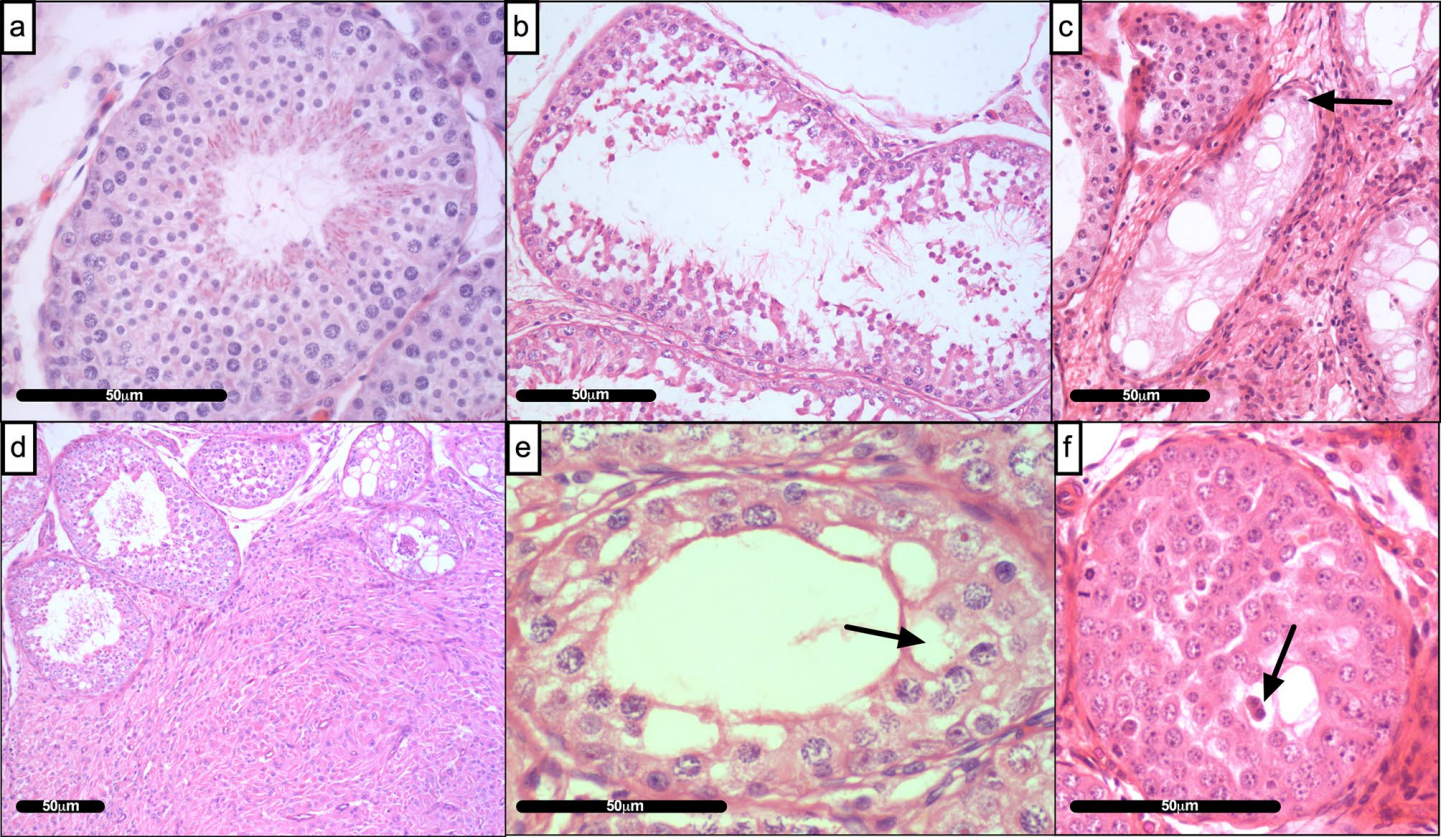
Histopathological features of dog testes. (**a**) Normal seminiferous tubule (**b**) Degeneration of the interstitium—Luminal cellular debris within tubule; (**c**) Sertoli cell only tubule; (**d**) Interstitial fibrous hyper-cellularity (**e**) Vacuolation of germ cells within a seminiferous tubule and (**f**) Multinucleated cells. Black arrows denote examples of detailed pathology observed. Scale bar represents 50 µm.

**Figure 2:**
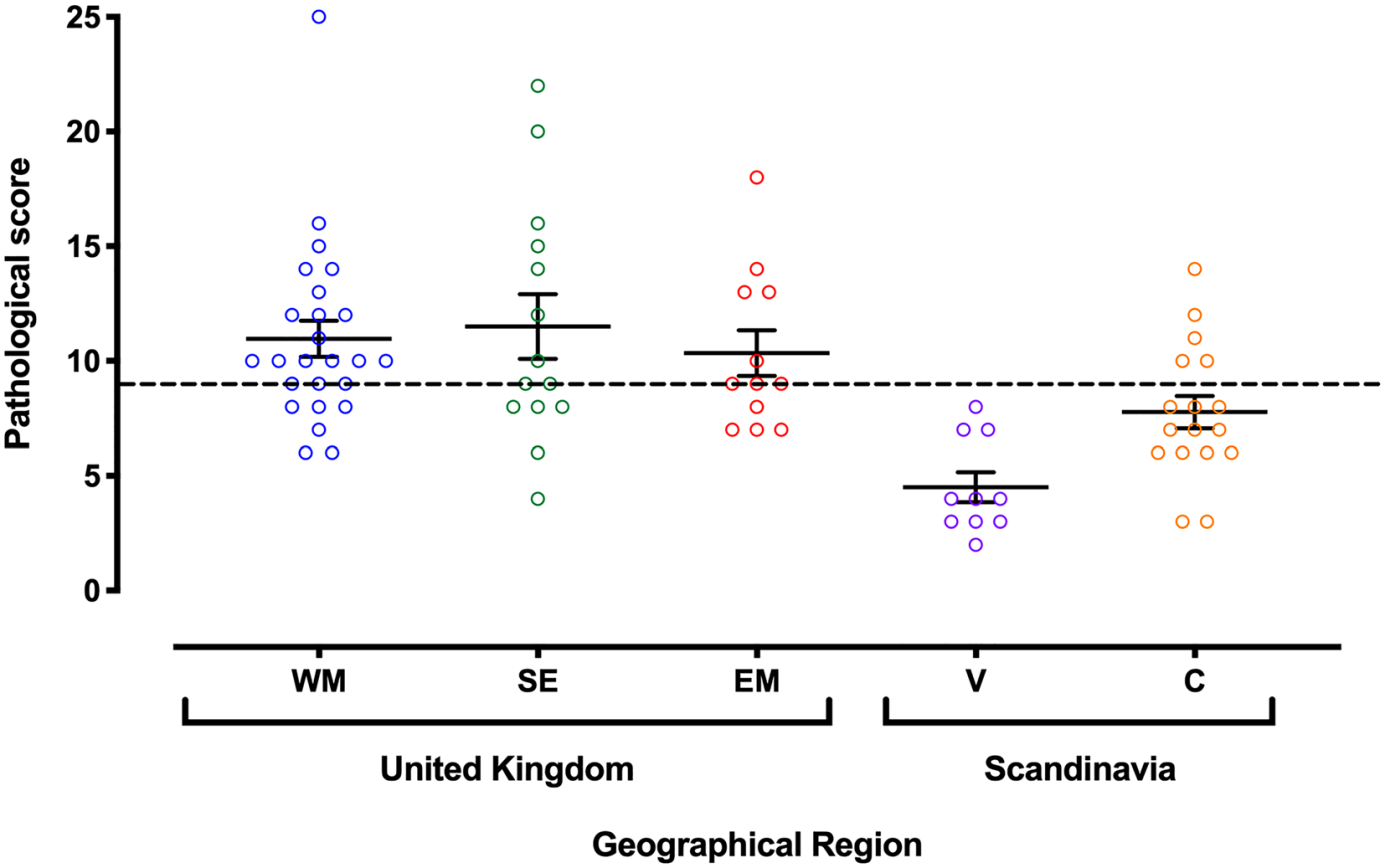
Regional differences in histopathology score of dog testes collected from five geographical regions. Each point represents a different testis from a specific geographical region; West Midlands, UK (WM: blue), South East, UK (SE: green), East Midlands, UK (EM: red), Vantaa, Finland (V: purple) and Copenhagen, Denmark (C: orange). Dotted line represents a base line histopathological abnormality characteristic of a normal testis calculated from the median value of all histopathological scores [n = 77]. Pathology score values above this line were considered abnormal. Error bars and lines denote mean ± 1 S.E.M. Figure was created using GraphPad Prism version 8.0 for Mac, GraphPad Software, California, USA (https://www.graphpad.com).

Examination of all haematoxylin & eosin (H & E) stained testis sections revealed a range of pathologies across geographic locations including; the presence of luminal cellular debris, Sertoli cell-only tubules, interstitial fibrous hyper-cellularity (possibly Leydig cell hyperplasia), vacuolated germ cells and multinucleated cells (Fig. 1, Table 1). The histopathological scoring of H & E stained testes sections from five geographical locations (UK: West Midlands, South East, East Midlands; Finland: Vantaa, Denmark: Copenhagen) revealed a significant difference in the incidence of testicular abnormalities (Fig. 2; p ≤ 0.0001). Testes from Finland had a significantly lower pathology score than all three UK regions; West Midlands [p ≤ 0.001], South East [p ≤ 0.001] and East Midlands [p ≤ 0.01]. Testes from Denmark were not significantly different to those collected in the UK but exhibited a trend towards reduced testicular health (Fig. 2).

**Table 1:** Overview of testicular pathologies by geographical region.

Region	Gross appearance	Tubular MNC	Interstitial MNC	Atrophic tubules	Degeneration	Luminal debris	Vacuolation	SCO tubules
WM	1.88 ± 0.43^ac^	1.16 ± 0.34^a^	0.44 ± 0.35	1.40 ± 0.37^ab^	1.76 ± 0.45^abc^	2.20 ± 0.35^a^	1.48 ± 0.43^a^	0.64 ± 0.34
EM	2.25 ± 0.58^ac^	0.67 ± 0.31^ab^	0.25 ± 0.22	1.75 ± 0.58^a^	2.17 ± 0.45^ab^	1.08 ± 0.32^ac^	1.25 ± 0.36^a^	0.92 ± 0.72
SE	2.50 ± 0.59^a^	0.79 ± 0.43^ab^	0.43 ± 0.25	1.50 ± 0.56^ab^	2.64 ± 0.64^a^	2.14 ± 0.70^ac^	0.71 ± 0.29^ab^	0.79 ± 0.69
V	**0.70 ± 0.23**^**b**^	0.30 ± 0.23^b^	0.30 ± 0.23	0.60 ± 0.24^b^	1.20 ± 0.30^bc^	**0.80 ± 0.20**^**b**^	0.30 ± 0.23^b^	0.30 ± 0.23
C	1.41 ± 0.39^bc^	0.71 ± 0.23^ab^	0.59 ± 0.25	1.00 ± 0.42^ab^	1.06 ± 0.40^c^	1.35 ± 0.24^bc^	1.24 ± 0.27^ac^	0.41 ± 0.39

Data denotes the mean pathology count ± 1 S.D; SCO = Sertoli cell-only; MNC = Multinucleated cells. Differences between superscripts depict significant differences. Bold depicts Vantaa Finland region significantly different to all three UK areas. See text for differences between UK areas.

Quantification of specific testis pathologies revealed further geographical differences between those originating from the UK and Scandinavia (Table 1). Testes from Finland had lower pathology scores for most pathologies: ‘gross appearance’ (overall histological abnormality), p ≤ 0.05 than all UK areas combined; fewer tubular multinucleated cells (p ≤ 0.05); fewer atrophic tubules than the South East, UK (p ≤ 0.05); less luminal debris than each of the three UK regions (West Midlands: p ≤ 0.001, East Midlands and South East: p ≤ 0.01) and less vacuolation (West Midlands: p ≤ 0.001, East Midlands: p ≤ 0.01). Testes from Denmark also generally had lower pathology scores than UK areas: South East (gross appearance: p ≤ 0.05; degeneration: p ≤ 0.001), East Midlands (degeneration: p ≤ 0.05) and West Midlands (luminal debris: p ≤ 0.001). Testes from Finland also had fewer vacuolated cells than those from Denmark (p ≤ 0.05). No other differences were noted between the two Scandinavia locations (Table 1).

### Localisation of Sertoli cells, germ cells and testicular cells undergoing proliferation

**Figure 3:**
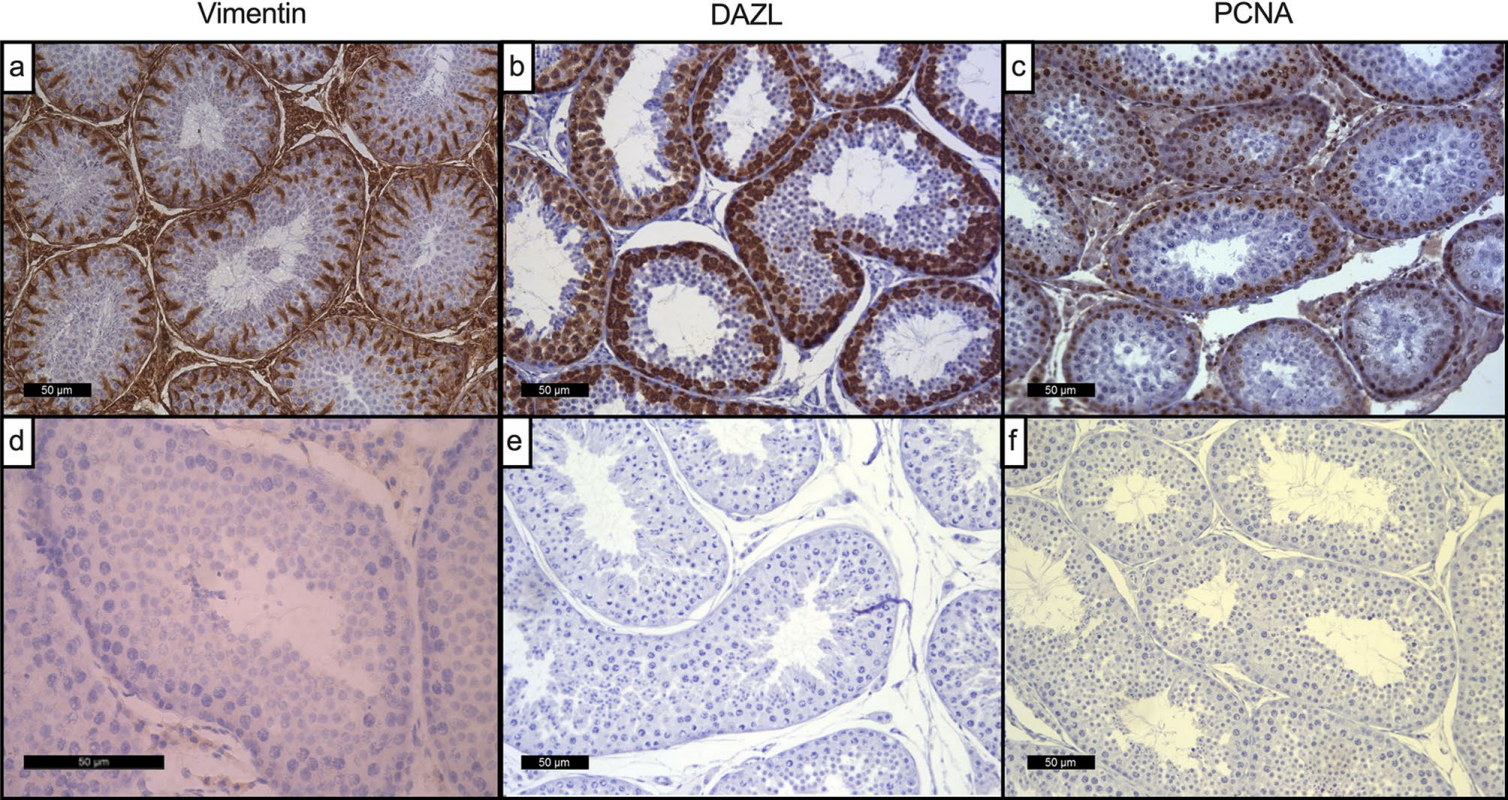
Immunohistochemical staining of mature dog testes for vimentin, DAZL and PCNA. Intense diffuse nuclear staining of Sertoli cells was observed for vimentin (**a**) and spermatogonia for both DAZL (**b**) and PCNA (**c**). Images (**d**–**f**) depict respective IgG negative controls. Scale bar represents 50 µm.

Figure 3 depicts examples of immunohistochemical staining of mature dog testes for vimentin, depleted in azoospermia-like protein (DAZL) and proliferating cell nuclear antigen (PCNA). Vimentin positive cells were localised across the seminiferous epithelium with intensity being primarily located to the basal region populated by Sertoli cells (Fig. 3a). DAZL was localised to primary spermatocytes and spermatogonia (Fig. 3b) whilst proliferating cell nuclear antigen (PCNA), a nuclear protein localised to sites of on-going DNA replication, was localised primarily to spermatogonia, lining the basement membrane (Fig. 3c). No staining was observed on negative control testis sections incubated with appropriate non-specific IgG antibodies (Fig. 3d–f: IgG controls for vimentin, DAZL and PCNA respectively).

### Regional variations in Sertoli cell numbers

**Figure 4:**
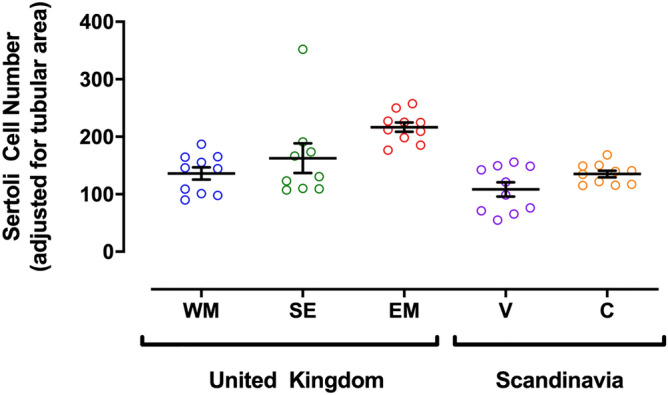
Regional variation in Sertoli cell numbers adjusted for tubular area. Sertoli cells were identified by vimentin immunohistochemistry. Each point represents an individual testis from a specific geographical location: UK: West Midlands (WM, blue), South East (SE, green), East Midlands (EM, red); Scandinavia: Vantaa, Finland (V, purple) and Copenhagen, Denmark (C, orange). Error bars and lines denote mean ± 1 S.E.M. Figure was created using GraphPad Prism version 8.0 for Mac, GraphPad Software, California, USA (https://www.graphpad.com).

Sertoli cell numbers, adjusted for tubular area, were calculated for each of the UK and Scandinavian locations. Figure 4 illustrates that testes from both Scandinavian locations and those from the West Midlands UK, had fewer Sertoli cells that those collected from the East Midlands [Vantaa: p ≤ 0.0001, Copenhagen: p ≤ 0.01, West Midlands: P ≤ 0.01, South East: NS). Eight of nine dog testes from the South East also had fewer Sertoli cells than the mean of the East Midlands cohort.

### Regional variation in testicular immunostaining for vimentin (Sertoli cells), deleted in azoospermia-like-protein (DAZL: germ cells) and proliferating cell nuclear antigen (PCNA)

**Figure 5:**
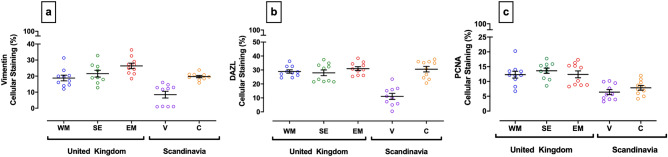
Quantification of immunostaining for vimentin (Sertoli cells), DAZL (germ cells) and a marker of cellular proliferation (PCNA) in dog testes from different geographical locations. (**a**) Vimentin, (**b**) DAZL, (**c**) PCNA. Each point represents an individual testis from a specific geographical region: UK; West Midlands (WM: blue), South East (SE: green) East Midlands (EM: red) and Scandinavia; Vantaa, Finland (V: purple) and Copenhagen, Denmark (C: orange). Error bars and lines denote mean ± 1 S.E.M. Figures were created using GraphPad Prism version 8.0 for Mac, GraphPad Software, California, USA (https://www.graphpad.com).

Figure 5 depicts the percentage cellular testicular area immunostained with antibodies against vimentin, DAZL and PCNA. For Sertoli cell staining (Fig. 5a), Finland was found to have significantly less vimentin staining than the UK regions, South East [p ≤ 0.05] and East Midlands [p ≤ 0.0001], and Denmark [p ≤ 0.05]. Testes from Vantaa also expressed a significantly lower percentage area stained for DAZL (Fig. 5b) than all other regions [p ≤ 0.05]. For cellular proliferation (Fig. 5c), PCNA immunostaining of germ cells (Fig. 3c) was significantly lower in testes from Finland compared to all three UK regions; West Midlands (p ≤ 0.05), South East (p ≤ 0.001) and East Midlands (p ≤ 0.01). Dog testes from Denmark also had significantly less PCNA staining than the South East (p ≤ 0.05).

### Regional variation in testicular chemical profiles

**Figure 6:**
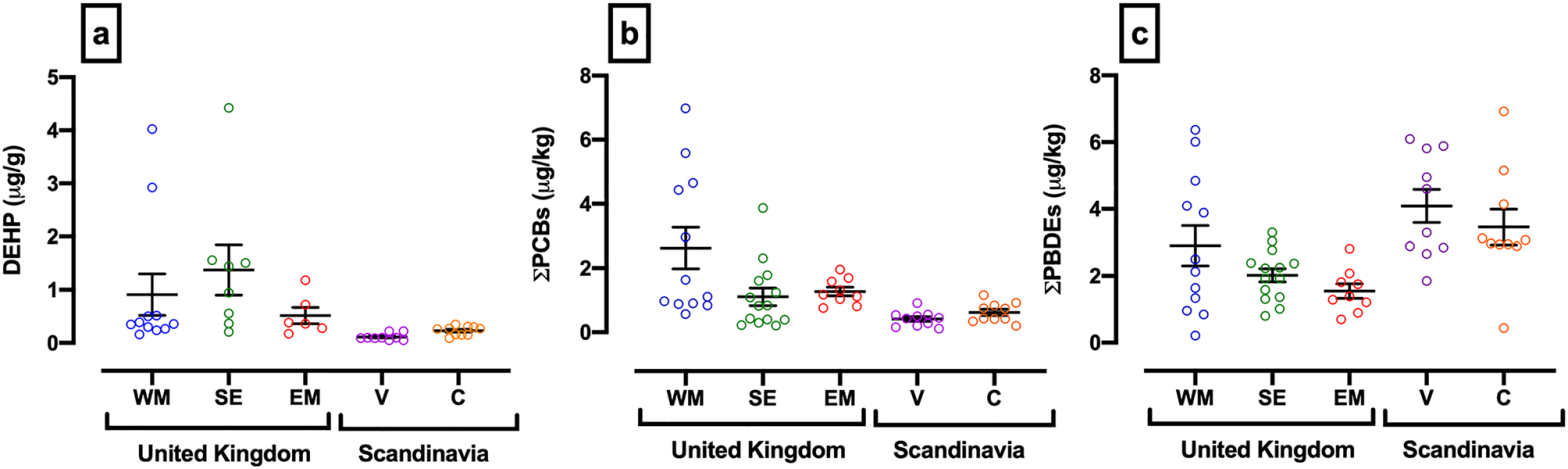
Mean testis concentrations of DEHP, ∑PCB congeners and ∑PBDE congeners across five national and international geographic boundaries: United Kingdom (West Midlands, South East and East Midlands), Finland (Vantaa) and Denmark (Copenhagen). DEHP (**a**), ∑PCBs (**b**; Mean concentrations of the sum of seven congeners of PCB) and ∑PBDEs (**c**; Mean concentrations of the sum of seven congeners of PBDE). Each point represents an individual testis from a specific geographical region: UK; West Midlands (WM: blue), South East (SE: green), East Midlands (EM: red) and Scandinavia; Vantaa, Finland (V: purple) and Copenhagen, Denmark (C: orange). Error bars and lines denote mean ± 1 S.E.M. Figures were created using GraphPad Prism version 8.0 for Mac, GraphPad Software, California, USA (https://www.graphpad.com).

The chemical concentrations of diethylhexyl phthalate (DEHP), sum of polychlorinated biphenyl congeners (PCB: 28, 52, 101, 118, 138, 153, 180) and sum of polybrominated diethyl ether congeners (PBDE: 28, 47, 99, 100, 153, 154, 183) found in the testes of dogs living in the five different geographical regions are illustrated in Fig. 6. A significant difference in chemical profile by region was observed (p ≤ 0.01). Testicular concentrations of DEHP were lower in Finland than in all three UK regions (Fig. 6a: West Midlands: p ≤ 0.01; South East p ≤ 0.0001; East Midlands: p ≤ 0.05). Testicular concentrations of DEHP were also lower in Denmark than in the South East (p ≤ 0.05). Concentrations of ƩPCB congeners (Fig. 6b) were greatest in the West Midlands, with significant differences between this region and; the South East (p ≤ 0.05), Finland (p ≤ 0.001) and Denmark (p ≤ 0.01). Concentrations of ƩPBDE congeners (Fig. 6c) were greatest in Finland, significantly higher than in testes from the South East and the East Midlands of the UK (p ≤ 0.01). Dog testes from Denmark also had significantly higher concentrations of ƩPBDE congeners compared to the East Midlands, UK (p ≤ 0.05).

**Figure 7:**
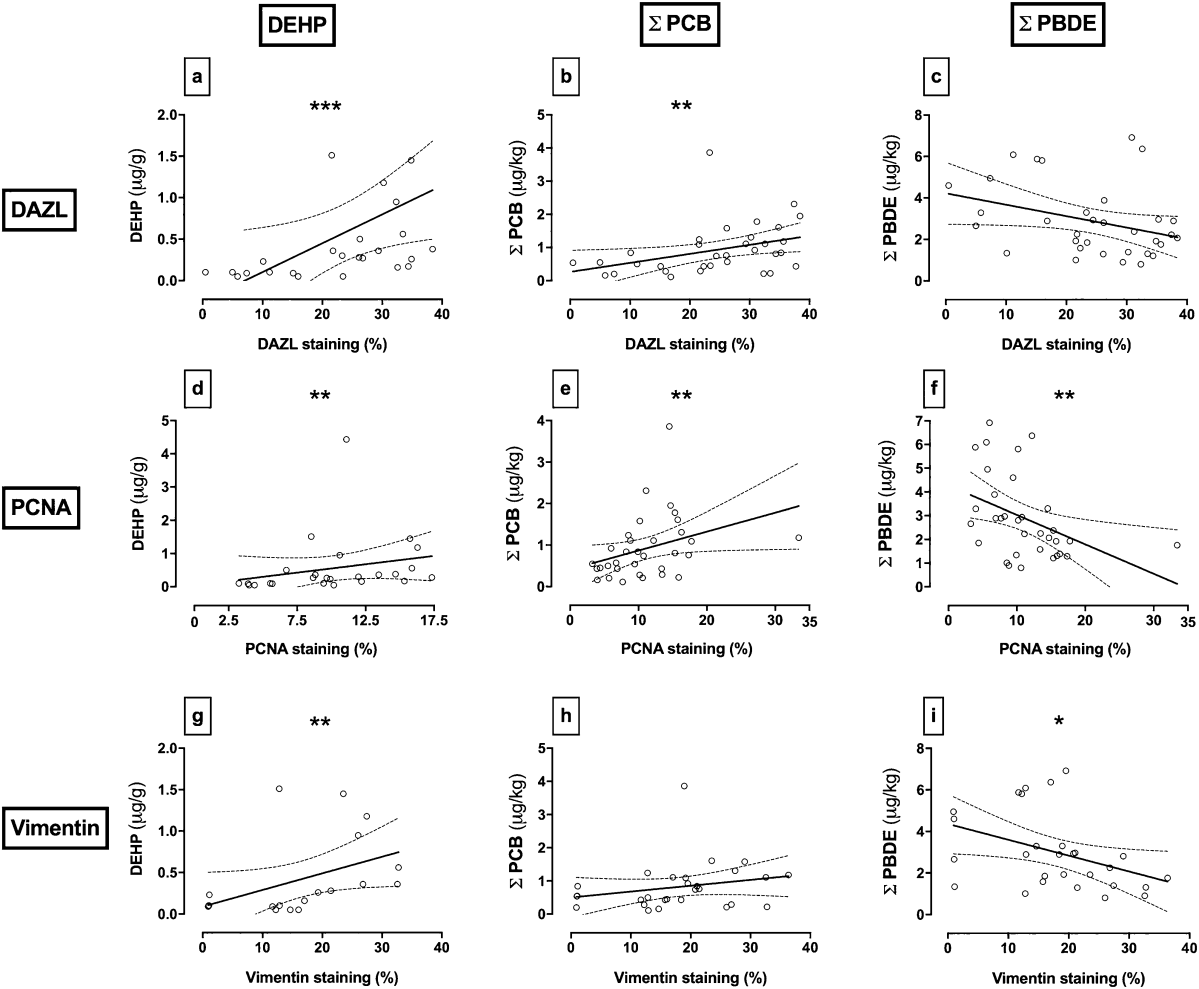
Associations between measurements of testicular chemicals, testis cell types and proliferation. Testis cell markers were identified by immunohistochemistry: Sertoli cells (vimentin), germ cells (DAZL) and proliferating cells (PCNA). **DAZL**: (**a**) Testicular DEHP [p ≤ 0.001; r = 0.6216; n = 25], (**b**) Σ PCB Congeners [p ≤ 0.01; r = 0.4676; n = 33] and (**c**) Σ PBDE congeners [p ≥ 0.05; r = − 0.2953; n = 33]. **PCNA**: (**d**) Testicular DEHP [p ≤ 0.01; r = 0.5592; n = 25], (**e**) Σ PCB congeners [p ≤ 0.01; r = 0.4628; n = 33] and (**f**) Σ PBDE congeners [p ≤ 0.01; r = − 0.4952; n = 33]. **Vimentin**: (**g**) Testicular DEHP [p ≤ 0.01; r = 0.622; n = 19], (**h**) Σ PCB congeners [p ≥ 0.05; r = 0.3583; n = 28] and (**i**) Σ PBDE congeners [p ≤ 0.05; r = − 0.4574; n = 28]. Each point represents an individual measurement from varying geographical regions. Line of best fit and 95% confidence band plotted for graphical representation only. * denotes significance level; p ≤ 0.05 [*]; p ≤ 0.01 [**]; p ≤ 0.001 [***]. Figures were created using GraphPad Prism version 8.0 for Mac, GraphPad Software, California, USA (https://www.graphpad.com).

Figure 7 depicts the relationship between the immuno-expression of vimentin, PCNA and DAZL, with the testicular concentrations of ΣPCB congeners (µg/kg), ΣPBDE congeners (µg/kg) and DEHP (µg/g). DAZL (germ cells) positively correlated with DEHP (Fig. 7a; p ≤ 0.001; r = 0.6216; n = 25) and ΣPCB congeners (Fig. 7b; p ≤ 0.01; r = 0.4676; n = 33) but showed no significant correlation with ΣPBDE congeners (Fig. 7c). PCNA positively correlated with DEHP (Fig. 7d; p ≤ 0.01; r = 0.5592; n = 25) and ΣPCB congeners (Fig. 7e; p ≤ 0.01; r = 0.4628; n = 33), but negatively correlated with the ΣPBDE congeners (Fig. 7f; p ≤ 0.01; r = − 0.4952; n = 33). Vimentin positively correlated with DEHP (Fig. 7g; p ≤ 0.01; r = 0.622; n = 19), negatively correlated with the ΣPBDE congeners (Fig. 7i; p ≤ 0.05; r = − 0.4574; n = 28) but showed no significant correlation with ΣPCB congeners (Fig. 7h). In addition, vimentin as a marker of Sertoli cells showed a strong positive correlation with DAZL as a marker of germ cells (p < 0.0001; r = 0.7452). Notably, negative or positive correlations of pollutants on Sertoli cells are paralleled by those observed on germ cells.

## Discussion

Data presented in this paper are significant since they illustrate for the first time that dog testes collected from different geographical locations in the UK and Scandinavia exhibit differences in (1) indices of testicular pathology, (2) Sertoli cell numbers, (3) indices of spermatogenesis and cellular proliferation and (4) testicular chemical profiles. Furthermore, our data indicate that the ΣPBDE congeners negatively correlate with Sertoli cell numbers and proliferative activity primarily in germ cells. Intriguingly, some chemical types positively correlated with DAZL (DEHP, ΣPCB), vimentin (DEHP) and PCNA (DEHP, ΣPCB). While we recognise that these association studies do not document cause and effect, it is tantalising to hypothesise that chemicals detected within testicular tissue are positioned to impact locally on testis function. Since the variation in testicular chemical profiles parallels our observations of altered or perturbed testicular morphology, we postulate that this may underlie reports of geographical variation in male dogs reproductive development and function.

The current study builds on our previous work in which we reported that temporal changes in male dogs reproductive function in a population of stud dogs from a controlled breeding programme, paralleled that reported in the human^16^. Specifically, this was manifest by a decline in semen quality over a 26-year period and male pups from the same population showed an increased incidence of cryptorchidism^16^. We further demonstrated that testes collected from dogs in the same area contain environmental contaminants and that testicular concentrations of these chemicals can adversely affect sperm function in short term cultures^17,18^. These data suggest that the dog may be a sentinel species for human exposure to contaminants and that reported geographical differences in human male reproductive function may be reciprocated in the dog. Here we have extended our work by assessing chemical profiles and morphology in dog testes collected from different UK locations, Denmark and Finland where differences in human male reproductive health have been reported^6^.

In the human, geographical differences have been reported for three major indices of male reproductive function: reduced sperm counts, increased incidence of testicular germ cell cancer (TGCC) and malformations of male infants at birth (hypospadias and cryptorchidism). This has been the topic of many independent studies and many extensive review articles^6,19^. Of note is that Denmark has been reported to have a 300% higher rate of testicular cancer compared to Finland, reduced semen quality and a higher rate of reproductive abnormalities^6,20–22^. In the dog, reports on the prevalence and incidence of testicular cancer are more limited than in the human. This likely reflects the fact that so many dogs are neutered thus restricting the population that could be monitored in a longitudinal study. Despite this limitation, it has been reported that the prevalence of testis cancer has increased from 16% in 1962 to 27% in 2007 and that some dogs exhibit the GCNIS precursor cells described in the human^23,24^.

In the current study, we have used testicular morphology and chemical profiles as a possible index of altered male reproductive function and/or health in the dog. Notably, we report that dog testes from Finland were different to those from Denmark and the UK in terms of reduced pathology. Although this parallels human studies indicating a lower relative prevalence of testicular cancer in Finland (vs Denmark), it is uncertain if the reduced testicular area occupied by Sertoli and germ cells in the Finnish dog samples equates to a relative difference in sperm quality. Indeed, extrapolating histological changes in the testis to sperm quality in adult dogs would be too much of a leap to make at this stage, particularly as temporal trends in the human and dog are manifest differently: reduced sperm counts in the human (reported as concentrations rather than total sperm output) versus reduced motility in the dog^3,16^. In the current study, the mechanisms underlying the differences in Sertoli and germ cell testicular area stained are uncertain. One possibility is that this reflects exposures during the fetal stage when Sertoli cell proliferation occurs. Indeed, it is during this period that exposures to environmental factors have been associated with perturbed development in adult life^4^. However, having demonstrated regional differences in contaminants and histological/pathological differences, this remains a focus for future studies in this species.

Notwithstanding, we do postulate that the pathological and histological differences observed in the current study may reflect differential environmental exposures based on regional differences that existed between 2014 and 2016 when the testes were collected. Furthermore, the concept of temporal changes in environmental linked pathologies is not without precedent: the incidence of human testicular cancer in Finland, Norway and Sweden is increasing whereas that in Denmark and Iceland has not changed since the 1990s^25–29^.

In the human, studies suggestive of a linkage between chemical exposure and perturbed male reproductive function have been largely epidemiological. Chemical concentrations in blood, breast milk, urine and to a lesser extent, semen, have been used as an index of environmental exposure^30,31^. For example, human serum PBDE concentrations have been associated with reduced sperm motility and this was linked to congener PBDE-47: the predominant PBDE congener in dog testes^16,32^. Furthermore, geographic differences in human blood and breast milk PBDEs have been attributed to differences in diet and exposure within dust^33^. In the current study, dog testes used as an index of environmental exposure have shown higher concentrations of PBDE congeners in dogs from Finland and a negative correlation with proliferating germ cell numbers. Although cause and effect is not conclusive, the difference between Scandinavian and UK testicular PBDE concentrations are striking and add to the weight of evidence linking environment to reproductive health. Differences in testicular DEHP and PCB concentrations across regions were similarly most evident in samples from Finland.

Our chemical analyses focussed on only three chemical types. This is not reflective of real-life exposure to chemical mixtures many of which interact and are influenced by metabolism and physico-chemical properties such as lipophilicity. Here we sought to demonstrate proof of concept by focussing on a selection of environmental contaminants previously identified in dog testis and shown to impact on sperm function^16–18^. Whilst many animal models have been used to demonstrate chemical effects, few are applicable to be used as real-life models, which approximate human exposure to chemical mixtures. One such model involves grazing sheep on pastures fertilised with processed human sewage sludge (biosolids) known to contain a wide range of anthropogenic chemicals at low levels. Notably, a cohort of male ram lambs from ewes exposed throughout pregnancy and then exposed post-weaning exhibited testicular abnormalities similar to those described in the current study: reduced germ cell numbers and Sertoli cell only tubules^10^. Reduced Sertoli and germ cell numbers have also been reported in late gestation fetuses and this is viewed as a presage to altered development and reproductive health in adult life^9^.

In conclusion, the dog has been used as a sentinel species to approximate human exposure to a selection of chemical mixtures present in the environment, including the household. Regional influences on human male reproductive health have been linked to differential chemical exposures, therefore geographical differences in testicular chemical content in the dog may also impact on male reproductive health. Although we recognise that correlating testicular chemical content with morphology does not demonstrate cause and effect, we propose that the regional differences in testicular pathology and histology add further support for an environmental influence on male reproductive function.

## Methods

### Processing of testis

Whole dog testes, collected from specific UK and Scandinavian geographical locations, were obtained as surplus material from routine castrations performed at veterinary clinics. All testes were collected from 2014 to 2016. To determine eligibility and inclusion criteria of the specimens, information was collected on dog breed, age at castration, background and health. This included clinical history (e.g. reason for castration, history of reduced fertility, other reproductive problems, disease, ill-health) dietary information and lifestyle factors. Key inclusion criteria included general good health and no history of fertility/reproductive problems. Breeds primarily included Labrador retriever, Golden retriever, German Shepherd, Curly coat retriever and Border Collie. Dog testis ages at castration comprised median (1.5 years), Q1 (0.77 years) and Q3 (4.29 years), with a range of 11.6 years. To ensure that testis tissues were handled and prepared in a similar way, irrespective of sampling location, attending veterinarians were provided with a protocol for testis collection and processing. For chemical analyses, testes were weighed, desiccated and stored at − 20 °C until analysed. For histological processing, both a diagram and instructions were provided to ensure that a 5 mm thick disk was cut from the centre of each testis and immediately immersed in Bouin’s fixative solution for six hours (Sigma-Aldrich). The testes disks were then transferred to 70% ethanol for storage until processing. All specimens were further processed into wax blocks at Nottingham. Testes were processed through a 17 h cycle in an automated Leica tissue processor (Leica Microsystems) and paraffin embedded. Five micrometre sections were cut using a fully automated rotary microtome (Leica RM2255; Leica Microsystems). Sections were transferred onto polysine slides (CAS: P4981; ThermoFisher Scientific Ltd) and dried overnight at 60 °C.

### Ethics

This research project was approved by the Committee for Animal Research and Ethics (CARE), University of Nottingham, School of Veterinary Medicine and Science [Refs: 208 101012, 513 120117 and 1097 140227]. Testes at all locations were collected only with full consent of the owners all of whom were provided with an overview of the research project. The consent and confidentiality form was consistent across clinics in the UK, Denmark and Finland. All testes collections were performed in accordance with relevant guidelines and regulations at each location. CARE requires that studies performed or coordinated in the UK have over-arching CARE ethical approval. Ethical approval granted by CARE was reviewed by the manager of the animal hospital at the University of Copenhagen and by the Finnish Guide Dog School. Since the consent process was consistent across all three sites, no further local ethical review was required.

### Pathology scoring

Dog testes originated from five locations [n = 77; West Midlands, UK = 25; South East, UK = 14; East Midlands, UK = 11; Copenhagen, Denmark = 17; Vantaa, Finland = 10]. Tissue sections were stained with haematoxylin and eosin and histopathological examination was undertaken by light microscopic examination. Testes were graded on a five-point scale for parameters indicative of abnormal pathology (depicted in Table 2). All testis scores were combined to calculate a median score and this was designated as the baseline against which abnormalities were defined. Since all normal heterogeneous tissues, in normal conditions, will exhibit some abnormal histopathological features, scores above this line were considered atypical. To control for processing artefacts, tissue handling was standardised across the five locations. Indices such as vacuolation and presence of luminal debris were therefore included as reported elsewhere^34^. In establishing the methodology RS and RL evaluated a cohort of sections for consistency after which all sections were evaluated by a single evaluator (RS) while blinded to the source of the testis. This ensured consistency with the scoring and avoided differences that may arise when data is combined from different evaluators.

**Table 2:** Evaluated testicular histopathological parameters.

Classification	Distinguishing feature	Grading scale	Magnification power
Sub-gross appearance	What is the overall appearance of testis?	Graded by 0–4 scale whereby 0 = typical and 4 = atypical	×100
Seminiferous tubules: evidence of atrophy or degeneration	Tubule size Loss of tubular cells Luminal diameter	0 = 0% 1 = 1–25% 2 = 26–50% 3 = 51–75% 4 = 76–100%	×400
Cellular changes: Sertoli cells Germ cells	Cytoplasmic vaculation Multi-nucleation Cell swelling Spermatogenic arrest GCNIS cells Cell sloughing Pyknosis Sertoli cell only		×400 or ×630
Interstitial changes	Multi-nucleation Fibrosis* Hypo-cellularity* Hyper-cellularity* Haemorrhage* Oedema*		×630 or ×100

The parameters were scored by a five-point scale and grading scale percentages relate to proportion of entire testis. Asterisk (*) denotes features assessed qualitatively and by general comment only.

### Immunohistochemistry

Ten dog testes from each location were immuno-stained for proliferating cell nuclear antigen (Abcam, [PCNA: ab18197]); deleted in azoospermia like factor (Santa Cruz Biotechnology Inc., [DAZL (C-20): sc-27333]) and vimentin (DAKO, [M7020]). Briefly, prepared slides were rehydrated and incubated in PBS. Heat induced epitope retrieval (HIER) was carried out by immersing the slides in 0.1 M sodium citrate buffer (PCNA and DAZL, pH 6) or TRIS–EDTA buffer (vimentin, pH 9). Slides were incubated in 3% H_2_O_2_ for 5 min to suppress endogenous peroxidase activity. Sections were briefly washed in PBS and incubated for 20 min in 1% normal blocking serum to block nonspecific binding of the biotinylated secondary antibody. Biotin blocking solution was applied for 15 min to block endogenous avidin/ biotin activity (CAS: SP-2001; Vector Laboratories Ltd, Peterborough, UK). PCNA and vimentin incubated tissues were labelled using the Vectastain Elite Universal avidin–biotin-peroxidase detection kit (Vectastain Elite ABC HRP Kit; CAS no: PK-6200; Vector Laboratories Ltd) and incubated with 3,3′ – diaminobenzidine substrate (DAB; SK-4100, Vector Laboratories Ltd). DAZL immunolocalisation required biotinylated rabbit anti goat IgG (Code BA-5000; Vector Laboratories Ltd) secondary antibody due to species specificity and therefore utilised the Vectastain Elite Goat IgG peroxidase kit [CAS no: PK-6105; Vector Laboratories Ltd].

### Cellular staining quantification

Vimentin, PCNA and DAZL were quantified using the analytical computer package ‘Image pro plus 6.3’ (Media Cybernetics, Maryland, USA). Prior to application of the package, 40 microscopy images at 200× magnification were obtained of each stained testis section using a Leica DM5000B upright microscope (Leica, Milton Keynes, UK). Ten randomly selected images were collected from each pole of the section. Each of these images were then subjected to image analysis by which the area stained for each marker (vimentin, PCNA and DAZL) was measured. This was facilitated by computer recognition of colour specific pixels overlaid on the DAB chromogen brown stained antigen. This was then expressed as a percentage of haematoxylin stained nuclei, which was similarly selected using the software package. The percentage cellular area stained was then objectively calculated for each marker and values exported to Microsoft Excel for analysis.

### Sertoli cell number quantification

Sertoli cells were identified by vimentin staining. Cell numbers, adjusted for tubule area, were calculated using ‘Image pro plus 6.3’ (Media Cybernetics). Forty random images (630× magnification) were taken of each vimentin immunostained testis, 10 from each ‘compass’ pole of the testis section. This was deemed representative of the testis section^35^. For each image, the tubule area (pixels) and all Sertoli cells were counted. This area of the testicular tubules (pixels) was then transformed into a ratio based on the total pixel area of each image. This was multiplied by the Sertoli cell count to ensure consistency across samples: *Sertoli cell count* * *(total area/tubule area)*.

### Chemical detection

Adult dog testes (n = 77), were subjected to environmental chemical analysis in an ISO17025 accredited laboratory (James Hutton Institute, Aberdeen). The predominant contaminants routinely measured were diethylhexyl phthalate (DEHP: detection limit = 0.05 µg/g dry material), a range of polychlorinated biphenyl congeners (PCB: 28, 52, 101, 118, 138, 153, 180: detection limit = 0.02 µg/kg for all congeners) and a range of polybrominated diethyl ether congeners (PBDE: 28, 47, 99, 100, 153, 154, 183: detection limits for congeners 28, 47, 99, 100 = 0.02 µg/kg and for congeners 153, 154, 183 = 0.5 µg/kg)^16^.

### Statistical analysis

Statistical analysis was undertaken by either; Ordinary one-way ANOVA incorporating Sidak’s multiple comparisons test for parametric data or analysis of variance incorporating Dunn’s multiple comparison tests for non-parametric data. A non-parametric Spearman’s rank correlation statistical test was used to investigate correlation coefficients and statistical dependence between two variables. Statistical significance was determined when P ≤ 0.05. All statistics was undertaken using GraphPad Prism version 8.0 for Mac, GraphPad Software, California, USA (https://www.graphpad.com).
